# A Comprehensive Review of the Cardioprotective Effect of Marine Algae Polysaccharide on the Gut Microbiota

**DOI:** 10.3390/foods11223550

**Published:** 2022-11-08

**Authors:** Kit-Leong Cheong, Biao Yu, Jing Chen, Saiyi Zhong

**Affiliations:** 1 Guangdong Provincial Key Laboratory of Aquatic Product Processing and Safety, College of Food Science and Technology, Guangdong Ocean University, Zhanjiang 524088, China; 2 Department of Biology, College of Science, Shantou University, Shantou 515063, China

**Keywords:** cardiovascular diseases, marine algae polysaccharides, gut microbiota, metabolites, cardioprotective activity

## Abstract

Cardiovascular disease (CVD) is the number one cause of death worldwide. Recent evidence has demonstrated an association between the gut microbiota and CVD, including heart failure, cerebrovascular illness, hypertension, and stroke. Marine algal polysaccharides (MAPs) are valuable natural sources of diverse bioactive compounds. MAPs have many pharmaceutical activities, including antioxidant, anti-inflammatory, immunomodulatory, and antidiabetic effects. Most MAPs are not utilized in the upper gastrointestinal tract; however, they are fermented by intestinal flora. The relationship between MAPs and the intestinal microbiota has drawn attention in CVD research. Hence, this review highlights the main action by which MAPs are known to affect CVD by maintaining homeostasis in the gut microbiome and producing gut microbiota-generated functional metabolites and short chain fatty acids. In addition, the effects of trimethylamine N-oxide on the gut microbiota composition, bile acid signaling properties, and CVD prevention are also discussed. This review supports the idea that focusing on the interactions between the host and gut microbiota may be promising for the prevention or treatment of CVD. MAPs are a potential sustainable source for the production of functional foods or nutraceutical products for preventing or treating CVD.

## 1. Introduction

Cardiovascular disease (CVD) has become a major disease that threatens the health of human beings. CVD is a leading cause of death and morbidity worldwide. In China, approximately 40% deaths are attributed to CVD, which is higher than the cancer death rate or death rate from other diseases. CVD was a main cause of death in 2016, accounting for 45.50% in rural areas and 43.16% in cities [1]. The prevalence of CVD in China has been continuously increasing, and an upward trend will continue in the future. CVD includes coronary heart disease, heart failure, cardiomyopathy, hypertension, and stroke. The prevalence of CVD is also quickly rising in other developing nations (such as India and the African and Latin American continents), primarily due to diseases associated with atherosclerosis. In addition, viral and postinfectious diseases, which are also common in underdeveloped nations, have a negative impact on the heart and blood vessels [2]. In the USA, CVD is also the leading cause of mortality. Although the death rate of people with CVD has fallen over the previous few decades, nearly 2200 individuals died in the USA of CVD each day in 2016 [3]. According to estimates, 92.1 million people in the USA have at least one type of CVD, and by 2030, it is predicted that 44% of U.S. adults will suffer from more than one type of CVD [4]. As for Europe, Australia, and some developed countries in South America, the mortality rates of CVD are the lowest in the world. Despite this, CVD remains the main cause of death in Europe, approximately 42% and 52% for men and women each year [5]. The actual risk factors for CVD are becoming clearer, including high blood pressure, diabetes, obesity, high consumption of alcohol, inadequate physical activity, and unhealthy foods [6]. Frequent exposure to unhealthy foods, such as those high in sugar, fat, and salt, and a low consumption of dietary fiber leads to the gut microbiome’s dysbiosis. However, while still not fully understood, it has been recognized that the complex interactions between dietary fibers and the intestinal flora and gut-generated metabolites may play a significant role in CVD. There is increasing research attention to the gut microbiota as a strategy for the protection and treatment of CVD.

The human gut microbiome is a complex community predominantly found in the large intestine. Bacteria in the colon comprise hundreds of species and are present at levels of approximately 10^11^–10^12^ cells per mL of colonic content [7]. The majority of bacteria come from two phyla, Bacteroidetes and Firmicutes [8]. The gut microbiota is involved in degrading otherwise indigestible food components in the upper gastrointestinal tract, such as polysaccharides, into compounds that the host can absorb, and this process yields numerous functional metabolites, several of which have beneficial effects on the intestinal barrier and cardiovascular health. In addition, a rich and diverse gut microbiome leads to a well-balanced and healthy composition. A healthy gut ecosystem is critical to immune function and preventing disease development. Gut dysbiosis may play a role in a number of diseases; first and foremost are gastrointestinal diseases, which are the most studied, followed by those related to metabolic syndromes. There is also evidence that CVD is strongly correlated with the intestinal flora. Because of the complexity of the intestinal flora, researchers remain challenged to explore the roles of the intestinal flora and their functional metabolites in potential applications for the management of human health and CVD conditions.

Marine algae are far more abundant than other resources in the oceans. Marine algae contain a wide range of nutrients, including polysaccharides, proteins, peptides, amino acids, mineral salts, lipids, and polyphenols [9]. Among various nutrients, polysaccharides are the main constituents and key biological compounds in marine algae [10]. Lately, there has been an upsurge of interest in the pharmaceutical activities and potential applications of marine algal polysaccharides (MAPs). Several researchers have demonstrated that MAPs have anti-inflammatory, antioxidant, immunomodulatory, antitumor, antibacterial, and antidiabetic activities in vivo, in vitro, and in clinical experiments [11,12,13,14,15,16]. MAPs are not digested directly in our upper gastrointestinal tract but can be utilized through fermentation by gut microorganisms [17]. This process may improve the gut microflora profile (such as the composition, diversity, and richness) and stimulate the production of functional metabolites by commensal bacteria.

Most existing reviews on MAPs have focused on their preparation, biological activities, and related industrial applications. However, to our knowledge, little research has focused on the correlation between MAPs and CVD in relation to gut ecology. Due to the significance of the intestinal flora in modulating the pharmaceutical activities of MAPs, a comprehensive understanding of the interactions between MAP and the intestinal flora is a precondition for elucidation of the mechanisms of MAPs in the regulation of CVD. This review focuses on MAPs and their cardioprotective role in regulating the gut microbiome. In addition, to obtain a new insight of the structure–function relationship whereby the effect of MAP regulation on the gut microbiota can exert potent cardioprotective activities, this review also attempts to interpret the gut microbiota-involved mechanisms involving MAPs, along with the latest understanding of MAP catabolism by microbiota.

## 2. Structure–Function Relationship of MAPs to Cardioprotective Activity

**Figure 1:**
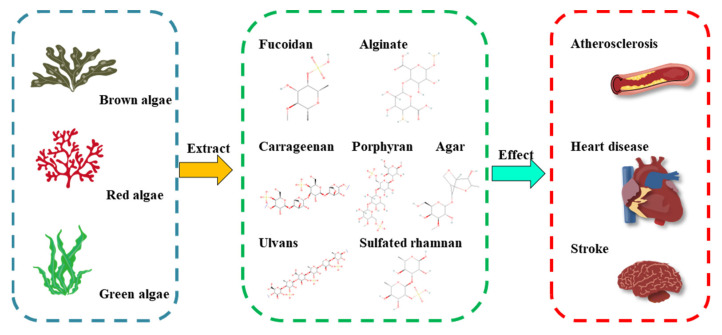
Applications of marine algal polysaccharides in CVD. Brown, red, and green algae contain different types of polysaccharides.

MAPs have unique large macromolecules that differ from intestinal plant polysaccharides and are also known as an ideal candidate for non-animal derived sulfated polysaccharides. Their chemical and physical properties vary according to the different algae varieties (Figure 1). The detailed structural features and protective effects of polysaccharides from different marine algae are listed in Table 1. For example, brown seaweeds (Phaeophyta) contain unique polysaccharides called fucoidan and alginates; red seaweeds (Rhodophyta) are composed of sulfated galactans such as carrageenans and porphyrin, whereas green seaweeds (Chlorophyta) consist of anionic polysaccharides such as sulfated rhamnans and ulvans. The nutraceutical and biological activities of MAPs usually result from the chemical and physical features, including the sulfation content, distribution of the sulfate groups, the monosaccharide composition, linkage type, molecular weight, and chain conformation. A basic understanding of the chemical and physical characters and cardioprotective activities is essential for successful MAP application in nutraceutical areas and will help access their multifunctional applications (Figure 1).

Fucoidan is a group of fucose-containing sulfated polysaccharides extracted from brown seaweeds. Fucoidan is mainly composed of L-fucose and sulfate groups, with a low percentage of other sugars such as xylose, glucose, galactose, mannose, rhamnose, arabinose, and glucuronic acid. They are linear polysaccharides and have a backbone built of α-(1→2)-linked fucose or α-(1→3)-linked fucose units with different substitutions [18]. Fucoidan structures and bioactivities differ among brown algal species. To investigate the relationship between fucoidan structural features and their endothelial protective activity, Chen et al. prepared four *Laminaria japonica* fucoidan fractions with different monosaccharide compositions, molecular weights, degrees of sulfatation, and sulfate positions [19]. They demonstrated that the fucoidan fractions with low-molecular (3.2 and 7.4 kDa) were more effective in the endothelial protection and downregulation of von Willebrand Factor, CD31, and CD51 expression in endothelial microparticles than the medium-molecular weight fucoidan fractions (28 and 34 kDa), while the highly sulfated fucoidan fractions (29.57% and 34.02%, respectively) were better at upregulating the fibroblast growth factor/FGF receptor signaling in BaF3 cells [19]. Alginate consists of two conformational isomer residues: (1,4)-linked β-D-mannuronic acid (ManA) and α-L-guluronic acid (GluA), which are arranged in an irregular blockwise pattern with different proportions of -GluA-GluA-, -ManA-GluA-, -ManA-ManA-, and -GluA-ManA- types [20]. Alginate is normally found in brown algae, following the genera *Laminaria*, *Saccharina*, *Ascophyllum*, *Durvillaea*, *Macrocystis*, *Ecklonia*, and *Lessonia* [20]. High-molecular weight sodium alginate significantly reduced the fasting blood glucose, homeostasis model values for assessment of insulin resistance, total cholesterol, and total body fat compared to the sodium alginate with a low molecular weight [21].

Agars, porphyrans, and carrageenans are other important sources of polysaccharides found in red seaweeds. Agar is composed of agarose and agaropectin, which are normally found in *Gelidium* and *Gracilaria* spp. The former is chemically composed of 3,6-anhydro-L-galactopyranose and D-galactose, whereas the latter is composed of alternating units of L-galactose and D-galactose linked by sulfate groups [22]. Agaropectin-derived oligosaccharides from *Gloiopeltis furcata* showed antidiabetic effects via the regulation of mitochondrial function and increased insulin sensitivity by regulating the reactive oxygen species (ROS)-mediated IRS/AKT/GSK-3β/GS signaling pathway [23]. Porphyran is usually extracted from the *Porphyra* species and is structurally similar to agarose, with alternating (1→4)-linked α-L-galactose 6-sulfate units and (1→3)-linked β-D-galactose units. Porphyran derived from the red seaweed *Porphyra haitanensis* as a food supplement has been demonstrated to protect against acute hyperlipidemia, as evidenced by the modulated lipid profile and increased levels of antioxidant enzyme activities in the liver, perhaps due to its antioxidant capacities [24]. Carrageenan is a group of sulfated polysaccharides formed by alternated units of D-galactose and 3,6-anhydro-D-galactose residues, which are connected through α-1,3-glucosidic and β-1,4-glycosidic linkages with sulfate ester units. According to the sulfate content and position, carrageenans are normally divided into six basic groups including Iota (ι)-, Kappa (κ)-, Lambda (λ)-, Mu (μ)-, Nu (ν)-, and Theta (θ)-carrageenan [25]. Valado et al. reported that carrageenan had a potential reduction in total cholesterol and low-density lipoprotein cholesterol (LDL-C) levels in the body, providing good protection against the development of CVD [26].

Ulvan and sulfated rhamnels are commonly isolated from green algae. Ulvan is a sulfated polysaccharide normally found in the green algae (Chlorophyceae) of the genera *Ulva* and *Enteromorpha* [27]. Ulvan is mainly built on disaccharides’ repeating sequences and is divided into two major types; one type is composed of *O*-3-sulfate rhamnose and β-D-glucuronic acid(1→4)-L-rhamnose 3-sulfate, while another is composed of *O*-3-sulfate rhamnose and α-L-iduronic acid(1→4)-L-rhamnose 3-sulfate [28]. The sulfate content of Ulvan could improve the antihyperlipidemic activity by improving the lipid profile and upregulating the expression of the farnesoid X receptor (FXR) and the peroxisome proliferators-activated receptor γ [29]. Low molecular weight (4.8 kDa) sulfated polysaccharides from *Enteromorpha prolifera* was shown to protect against hypoglycemia by modulating the gene expression of glycogen synthase kinase 3 (GSK-3), insulin receptors, insulin receptor substrate 2 (IRS-2), protein kinase B (PKB), and phosphatidylinositol 3 kinase (PI3K) [30]. Sulfated rhamnan with a backbone of →3)-α-L-Rhap-(1→ and →2)-α-L-Rhap-(1→ residues is another typical structure of green algae polysaccharides. Sulfated rhamnan purified from *Monostroma nitidum* showed marked thrombolytic activity and antithrombotic activity in vivo and in vitro, as investigated by plasminogen activator inhibitor-1, D-dimer levels, fibrin degradation products, and the rate of recanalization using a rat model of FeCl_3_-induced carotid artery thrombosis [31].

The cardioprotective effects of MAPs demonstrate that MAPs can be a good source of nutraceuticals for lifestyle diseases. Numerous studies have shown that the cardioprotective activities of MAPs include normalizing lipid metabolism, decreasing oxidative stress, influencing biomarkers of acute and chronic inflammation in immune cells, and inducing positive changes in immune response. The relationship between MAPs and intestinal flora can provide new insights into the beneficial effects of MAPs on CVD, which has attracted growing attention.

## 3. Cardioprotective Effect of MAPs Associated with Gut Microbiota Modulation

Predictably, gut microbiota dysbiosis is most often associated with gastrointestinal disorders, which in turn influence the host immune response. Emerging evidence suggests an interaction between the gut microbiome and CVD, including atherosclerosis, hypertension, peripheral artery disease, atrial fibrillation, myocardial fibrosis, and heart failure [32]. Many studies have reported alterations in the diversity and composition of the gut bacteria in humans with CVD, as summarized in Table 2. For example, the obesity component, which is a CVD risk factor, is involved in the development of dysbiotic gut microbiota, an imbalance favoring Firmicutes and Bacteroidetes in obesity in humans and animals [33].

Bacteroides species are anaerobic gut commensals in humans and are recognized as conferring a myriad of benefits to the human host. Among them is the provision of energy from a variety of MAPs that are known to produce several secondary metabolites that are beneficial to the intestinal mucosal layer and decorate the surface of other microbes [34,35]. Bacteroides species exhibit genome-encoded carbohydrate active enzymes (CAZymes), which can hydrolyze glycoside linkages by following the release of the reducing sugars from MAPs [36]. CAZymes have five classes of carbohydrases: glycoside hydrolase (GH), glycosyltransferase (GT), polysaccharide lyase (PL), carbohydrate esterase (CE), auxiliary activities, and carbohydrate-binding module (CBM). Nguyen et al. reported a significant increase in Bacteroides with higher energy metabolism due to a laminarin-supplemented high-fat diet [37]. They showed a higher abundance of CAZymes in laminarin-supplemented high-fat diet mice compared to high-fat diet mice, with especially dramatic increases in glycoside hydrolases and polysaccharide lyases, including GT2, GT4, GH2, CE8, CE12, PL1, and PL10. Porphyran from *Porphyra haitanensis* decreased lipid accumulation and maintained gut microbiota homeostasis in diet-induced obese mice, including an increase in the relative proportion of Bacteroides, Roseburia, and Eubacterium and a marked reduction in *Helicobacter*.

Firmicutes and Bacteroidetes represent greater than 90% of the total bacteria community, while the Firmicutes/Bacteroidetes ratio was reported to be increased in spontaneously hypertensive rats [33]. The Firmicutes/Bacteroidetes ratio has emerged as a possible characteristic for gut dysbiosis and CVD risk factors. A low Firmicutes/Bacteroidetes ratio was found to be strongly correlated with a balanced immune status and is generally considered beneficial for health [38]. Several studies have demonstrated that polysaccharides derived from natural resources efficiently reduced the Firmicutes/Bacteroidetes ratio. *Sargassum pallidum* polysaccharides modulated the gut microbiota composition by decreasing the ratio of Firmicutes/Bacteroidetes and enhancing the relative proportion of some beneficial genera, such as *Bacteroides*, *Dialister*, *Phascolarctobacterium*, *Prevotella*, and *Ruminococcus* when investigated by in vitro fermentation assay [39]. MAPs treatment can reduce the ratio of Firmicutes/Bacteroidetes, as verified in an in vivo CVD risk model. The green alga *Enteromorpha prolifera* polysaccharide showed anti-hyperuricemic effects, including significantly lowering the level of serum uric acid, xanthine oxidase, and blood urea nitrogen. *Enteromorpha prolifera* polysaccharide maintained the stability of the intestinal flora and showed a significant decrease in the Firmicutes/Bacteroidetes ratio [40].

An increase in the growth of *Akkermansia muciniphila* has been found to be favorable for the prevention of type 2 diabetes, obesity, atherosclerosis, and other metabolic syndromes. *Akkermansia* is the only genus of the phylum Verrucomicrobia found in gastrointestinal samples. In high-fat diet-induced obese mice, the abundance of *Akkermansia muciniphila* was strongly correlated with the expression of fat metabolism and inversely related with inflammation in adipose tissue and circulating glucose, adiponectin, leptin, triglycerides, and insulin [41]. Shang et al. reported that *Enteromorpha clathrata* polysaccharides dramatically elevated the relative abundance of *Akkermansia muciniphila*, *Bifidobacterium* spp., and *Lactobacillus* spp. in the gut [42]. In addition, they demonstrated a similar beneficial pharmacological effect of two fucoidans from *Laminaria japonica* and *Ascophyllum nodosum* on diet-induced metabolic damage in the C57BL/6 J mice model, which increased the abundance of *Akkermansia muciniphila*, *Alloprevotella*, *Blautia*, and *Bacteroides* treatment by fucoidan [43].

*Bifidobacterium* and *Lactobacillus* species are well-known probiotics that beneficially affect the host organism by improving and modulating the intestinal flora and preventing CVD [44]. *Bifidobacterium* and *Lactobacillus* strains possess different degrees of cholesterol removal from the media through cholesterol assimilation during growth, the binding capacity of cholesterol to cells, the incorporation of cholesterol into the cytoplasmic membrane, and bile salt deconjugation [45]. Alginate oligosaccharide treatment improved fat metabolism and inflammation by regulating the intestinal microbiota in high-fat diet-induced gut dysbiosis mice, especially by increasing the abundance of *Lactobacillus gasseri* and *Lactobacillus reuteri* [46]. *Undaria pinnatifida* polysaccharide intervention significantly reduced the fasting blood glucose levels, mitigated the impaired glucose tolerance, and improved insulin resistance in diabetic rats by promoting the growth of beneficial bacteria, such as *Bifidobacterium*, *Lactobacillus*, *Faecalibaculum*, *Lachnoclostridium*, and *Olsenella* [47].

**Figure 2:**
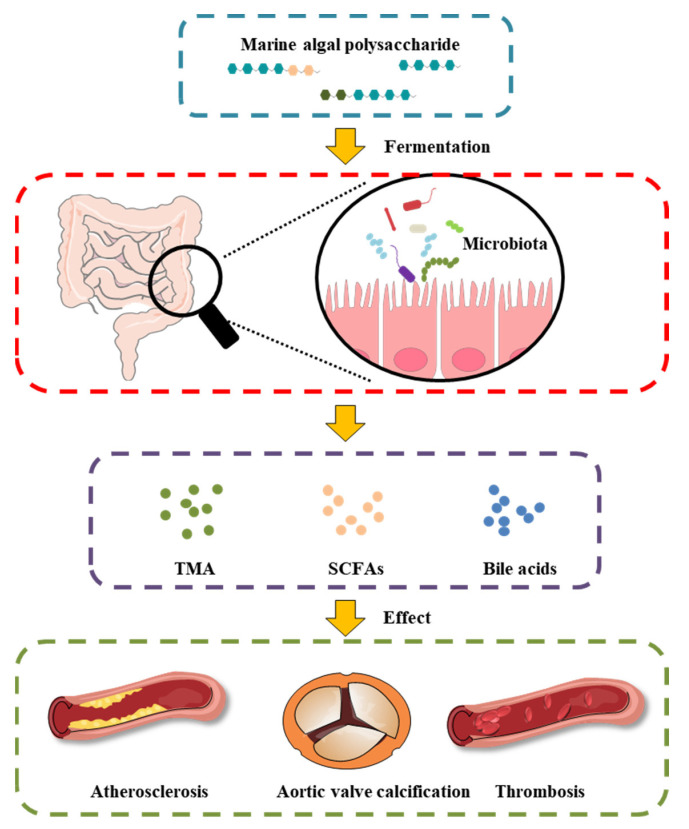
Effect of MAP on the intestinal microbiota and gut-derived metabolites in CVD.

MAPs play an important role in the intestinal microenvironment reconstruction, based on both richness and diversity. We suggest that the therapeutic manipulation of the intestinal microbiota with MAPs may be a successful treatment selection to restore gut microbiota composition and potentiate heart failure management (Figure 2). Undigested MAP can be fermented by intestinal microbiota for their growth and the production of secondary metabolites, such as SCFAs, bile acids, and TMA.

**Table 2:** Marine algal polysaccharides and their effect on CVD prevention through gut microbiota.

Type of Polysaccharides	Marine Algae Sources	Influence on Intestinal Microbiota	Treatment and Prevention of CVD	Ref.
alginate	*Sargassum fusiforme*	*Lactobacillus*, *Bacteroides*, *Akkermansia Alloprevotella*, *Weissella*, and *Enterorhabdus* ↑ *Turicibacter* and *Helicobacter* ↓	attenuated pathological changes in adipose, hepatic, and heart tissues; diminished oxidative stress	[48]
carrageenan	*Kappaphycus Alvarezii*	*Parasutterella*, *Alloprevotella*, *Oscillibacter*, *Melainabacteria*, and *Butyricimonas* ↑ *Clostridia*, *Erysipelotrichaceae*, *Blautia*, and *Lachnospiraceae* ↓	decreased total cholesterol and high-density level cholesterol; reduced adipocyte size and levels of adiponectin and leptin	[49]
fucan	*Saccharina japonica*	*Bacteroides sartorii*, *Bacteroides acidifaciens*, *Akkermansia*, and *Lachnospiraceae* NK4A136 ↑	prevented high-fat diet-induced obesity; regulated blood glucose/lipid metabolism	[50]
fucoidan	*Laminaria japonica*	phylum Bacteroidetes and families *Muribaculaceae* and *Bacteroidaceae* ↑	ameliorated high-fat diet-induced body weight gain, fat accumulation, serum lipid profiles, insulin resistance, hepatic steatosis, and adipocyte hypertrophy	[51]
fucoidan	*Sargassum fusiforme*	*Bacteroides*, *Faecalibacterium*, and *Blautia* ↑	reduced epididymal fat deposition, decreased oxidative stress, and attenuated the pathological changes in heart tissues	[52]
fucoidan	*Sargassum fusiforme*	*Bacteroides*, *Ruminococcaceae*, and *Butyricoccus*↑ *Helicobacter*↓	reduced fat accumulation; enhanced the energy expenditure through increasing the expression of uncoupling protein 1 in adipose tissues	[53]
porphyran	*Porphyra haitanensis*	*Roseburia* and *Eubacterium* ↑, *Helicobacter* ↓	ameliorated body fat accumulation in liver, serum, and adipose tissues; increased the pathway of PGC 1α-UCP 1-mitochondrial to produce more energy	[54]
porphyran	*Neoporphyra haitanensis*	*Parabacteroides* and *Coriobacteriaceae UCG-002* ↑	inhibited G6Pase and PEPCK enzymes related to hepatic gluconeogenesis; enhanced the expression of the GLUT4 enzyme involved in peripheral glucose uptake	[55]
ulvan	*Enteromorpha prolifera*	*Desulfovibrio* ↑, modulated *Verrucomicrobiaceae*, *Odoribacteraceae*, *Mogibacteriaceae*, *Planococcaceae*, and *Coriobacteriaceae*	decreased levels of inflammatory factors, including IFN-γ, TNF-α, and IL-6; increased total antioxidant capacity and superoxide dismutase, glutathione, catalase, and telomerase levels	[56]
ulvan	*Ulva lactuca*	*Dubosiella*, *Lactobacillus*, and *Parasutterella* ↑ *Staphylococcus*, *Escherichia−Shigella*, and *Ruminococcus* ↓	reduced the amount of blood urea nitrogen, serum uric acid, and creatinine; suppressed the activities of serum and hepatic xanthine oxidase	[57]

## 4. Effect of Gut Microbiota-Generated Short-Chain Fatty Acids in CVD

**Figure 3:**
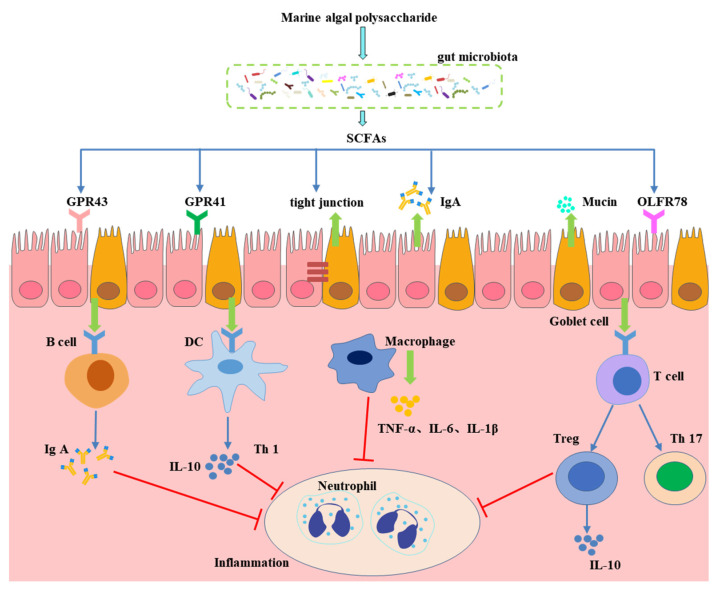
Functional impact of gut-derived SCFAs on host health. SCFAs are produced from the fermentation of MAP in the colon by gut microbiota.

Diet influences the intestinal microbiota by affecting the proportion and type of metabolites produced. Short-chain fatty acids (SCFAs) are the main products of saccharolytic fermentation of MAPs by the intestinal flora in the large intestine [17]. Acetate, propionate, and butyrate are the three major SCFA products and are generated at an approximate molar ratio of 60:20:20, producing an amount of over 100 mmol/L in the intestinal lumen [58]. There are many SCFA-producing organisms in the gut microflora, including *Alloprevotella*, *Bacteroides*, *Clostridium*, *Eubacterium*, *Faecalibacterium*, and *Roseburia* [59]. It is possible that the direct effect of modulating CVD risk is the SCFA regulation of blood pressure, increased lipid metabolism, energy depletion, and peptide YY release, while inhibiting the phosphorylation of adipose triglyceride lipase [60]. The most beneficial roles of SCFAs in the gastrointestinal tract are mediated by the direct activation of their receptor, the G protein-coupled receptor 41 (GPR41), also known as free fatty acid receptor 2 (FFAR3), and GPR43/FFAR2 (Figure 3) [61]. *Gracilaria lemaneiformis* polysaccharides was shown to alleviate high-fat diet-induced obesity by inhibiting fat accumulation in body organs, modulating gut microbiota dysbiosis, and improving lipid metabolism by increasing the concentration of SCFAs and the expression of GPR41 and GPR43 [62]. SCFAs can influence immune cells and increase energy expenditure. SCFAs upregulate the expression of MUC-related genes in goblet cells and tight junction-related protein genes to maintain the integrity of the gut barrier and decrease the risk of CVD.

Colonic acetate is produced by several commensal gut microbiota, such as *Bacteroides* spp., *Bifidobacterium* spp., and *Lachnospiraceae* spp., after dietary fiber utilization, which has beneficial effects on appetite regulation, fat oxidation, impaired glucose tolerance, improved inflammatory status, and attenuated cardiometabolism [63]. Butyrate is produced by butyrate-producing bacteria, such as *Eubacterium rectale*, *Faecalibacterium prausnitzii*, *Roseburia* spp., and *Clostridium* spp., and is an important fuel for intestinal epithelial cells [64]. Propionate is produced from polysaccharide fermentation by gut bacteria through the succinate pathway (most hexose and pentose sugars) and the propanediol pathway (deoxy sugars fructose and rhamnose). *Bacteroides* spp., *Clostridium* spp., *Propionibacterium* spp., *Dialister*, and *Veillonella* are common propionate producers [65]. Propionate was shown to directly promote satiety, improve pancreatic function, modulate hepatic lipid accretion, and reduce cholesterol levels [66]. Cooperation between different microorganisms in our diverse microbiota community to produce secondary metabolites is of interest to researchers. Zeybek et al. demonstrated substantial cooperation between *Bifidobacterium* and *Bacteroides* species in the fermentation of xylan-type polysaccharides to SCFAs [67]. Butyrate exhibited a wide variety of pharmaceutical activities in the treatment and management of CVD via different pathways, including energy homeostasis, glucose-fatty acid metabolism, inflammation process, oxidative stress, and neural signaling [68]. *Enteromorpha clathrata* polysaccharides improved intestinal dysbiosis caused by a high-fat diet, increased the abundance of *Eubacterium xylanophilum*, a butyrate-producing bacterium, and increased the level of butyrate [69].

A low fiber intake or a high-fat diet may not only damage the gut microbiota composition and diversity but also decrease the production and circulating levels of SCFAs. MAPs are a good source for increasing SCFA concentration, according to in vitro fermentation and in vivo experiments. In our previous studies, polysaccharides derived from *Gracilaria lemaneiformis* [34], *Porphyra haitanensis* [70], and *Saccharina japonica* [71] also showed significant increases in total SCFA production, particularly acetate, propionate, and butyrate, using in vitro fermentation with human gut microbiota. *G. lemaneiformis’* polysaccharides and oligosaccharides improved dextran sulfate sodium-induced colitis symptoms by modulating the gut microbiota and increasing the levels of total SCFAs and individual SCFAs [72,73].

## 5. Bile Acids as a Link between the Gut Microbiota and CVD

**Figure 4:**
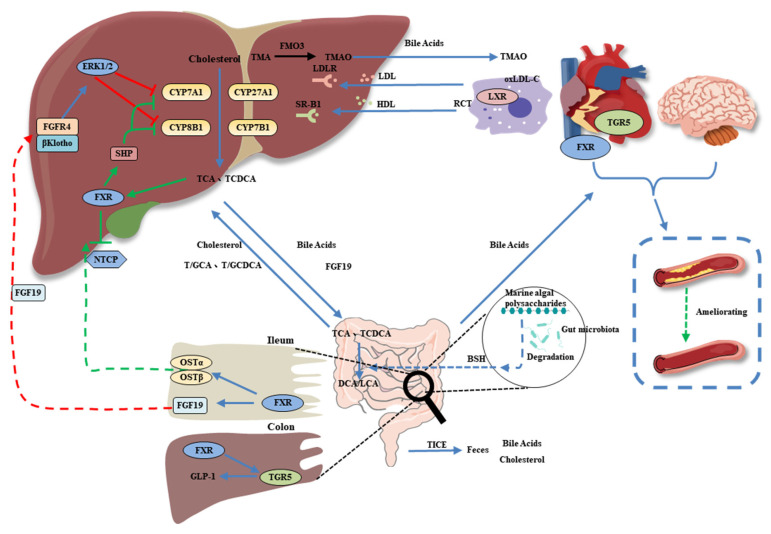
Effect of gut microbiota-derived bile acids on the CVD. Bile acids in turn impact the host physiology though interaction with FXR (farnesoid X receptor), LXR (liver X receptor), PXR (pregnane X receptor), and TGR5 (takeda G-protein–coupled receptor 5).

The second most primary metabolite of the gut microflora that is beneficial to CVD is bile acids. The functions of bile acids include the absorption of lipid-soluble nutrients, bile acid metabolism, energy expenditure, glucose homeostasis, lipid metabolism, gut motility, and immune cell regulation. They are recognized as signaling molecules through FXR, the pregnane X receptor (PXR), the vitamin D receptor (VDR), and the G protein-coupled receptor bile acid receptor 1 (TGR5) (Figure 4) [74]. Primary bile acids (chenodeoxycholic acid and cholic acid) are converted to over 50 different secondary bile acid metabolites by the gut microbiota. For example, cholic acid and chenodeoxycholic acid are synthesized in the liver and regulated by intestinal bacteria into secondary deoxycholic and lithocholic acids and a number of less abundant metabolites, which can all be reabsorbed from the intestine [75]. The ι-carrageenan tetrasaccharide hydrolyzed from ι-carrageenan increased the overall levels of bile acids in the serum, liver, and feces and alleviated liver lipid accumulation through the expression of bile acid-related receptors such as FXR and PXR signaling to regulate cholesterol conversion and fatty acid metabolism [76].

In the intestine, primary bile acids are catalyzed by a series of enzymatic reactions, such as deconjugation (bile salt hydrolase) and dehydroxylation (7-dehydroxylase), in which enzymes are secreted by the gut microbiota. Bile salt hydrolases have been characterized in species of *Bacteroides*, *Bifidobacterium*, *Clostridium*, *Enterobacter*, *Enterococcus*, and *Lactobacillus* [77]. Supplementation with fucoidan extracted from *Undaria pinnatifida* improved the serum total bile acid levels, aortic arch injury, gut microbiota modulation, and bile salt hydrolase activity in rats with dyslipidemia [78]. The bile acid metabolism-related pathway is correlated with bile salt hydrolase, which is produced by the intestinal microbiota that metabolizes host bile acids and regulates the rate-limiting enzyme of bile acid synthesis in the liver (CYP7A1) [78].

Yang et al. also found that fucoidan A2 from the brown algae *Ascophyllum nodosum* accelerated bile acid synthesis by improving the expression of CYP7A1 in high-fat diet mice [79]. Therefore, MAPs can modulate the abundance of bile salt hydrolase-producing bacteria and change bile acid composition to regulate signaling through bile acid-related receptors, thereby regulating human heart health.

## 6. MAP Modulates the Gut Microbiota-Derived Metabolite TMAO

**Figure 5:**
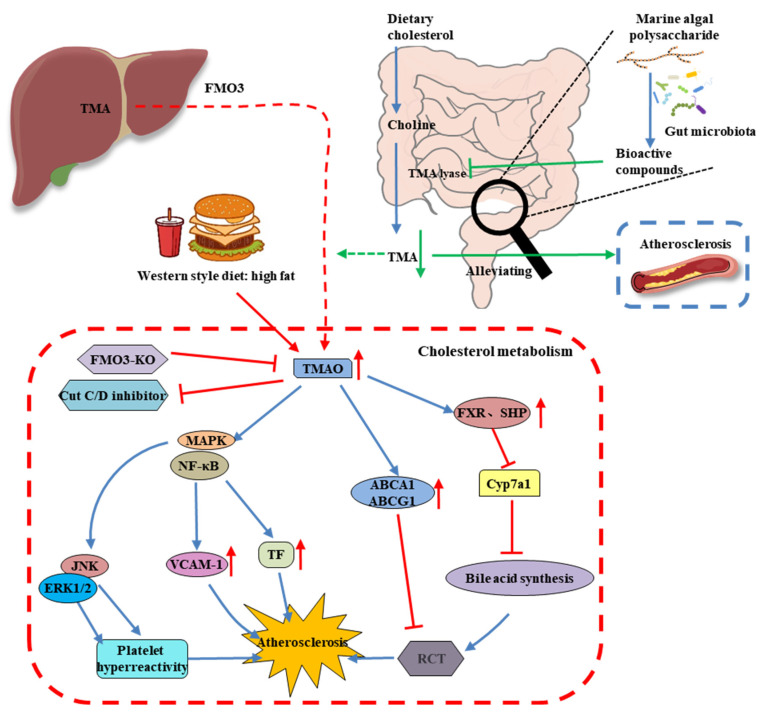
Dietary precursors, such as choline and L-carnitine, are metabolized into TMA by gut microflora through their related enzymes. Host hepatic FMO (flavin monooxygenases) oxidize TMA into TMAO, which promotes metabolic and functional changes in the host including CVD.

Trimethylamine-N-oxide (TMAO) is a key class of microbial-derived metabolites, derived from dietary choline, L-carnitine, betaine, and other choline-containing compounds by the action of gut microbiota as trimethylamine (TMA), which is further converted to TMAO by hepatic flavin monooxygenases (Figure 5) [80]. Recently, TMAO has emerged as a significant mediator and powerful prognostic marker. Evidence from experimental and clinical studies has demonstrated a close relationship between the gut microbiota and multiple CVDs, such as atherosclerosis, hypertension, and myocardial infarction [81]. The components of diets are a major factor that affects TMAO levels and CVD progression. Diets that are high in fat and animal proteins are associated with gut microbiota dysbiosis and upregulated plasma TMAO levels [82]. Therefore, dietary supplements and lifestyle interventions, especially probiotics, may markedly decrease TMA and TMAO levels by restructuring the gut microbiota.

Several gut microbiota are involved in TMA and TMAO formation, including Deferribacteraceae, Anaeroplasmataceae, Prevotellaceae, and Enterobacteriaceae [83]. *Chlorella pyrenoidosa* and *Spirulina platensis* polysaccharides restored the HFD-induced decrease in the relative abundance of gut bacterial TMA-producing enzymes and pathways [84]. *Sargassum fusiforme* fucoidan modulated the intestinal microbiota, especially by decreasing the proportion of Proteobacteria to decrease the metabolism of carnitine and choline, thereby reducing the formation of TMA and TMAO [52].

## 7. Conclusions and Future Perspectives

Increasing research has shown that modulation of intestinal flora composition and function plays a pivotal role in the prevention and treatment of CVD. Recent studies have provided evidence that MAPs are beneficial for human health and nutrition. MAPs have received significant attention as dietary polysaccharides for manipulating the gut microbiome.

This review summarized the beneficial effects of MAPs on CVD by balancing the gut microbiota. MAPs are inclined to increase the abundance of Bacteroides, Akkermansia, Bifidobacterium, and Lactobacillus, while reducing Firmicutes and the Firmicutes/Bacteroidetes ratio to prevent or treat CVD. In addition, the profitable effects on heart health seem to be modulated by the secondary metabolites generated by the intestinal microbiota. SCFAs have been shown to modulate the cell signaling pathways involved in metabolic homeostasis and immune responses. Second, the biotransformation of MAP bile salts by the gut microbiota affects CVD. MAPs also modulate the intestinal flora to influence TMA and TMAO metabolism.

In the future, we expect in-depth studies to provide the basis for the development of MAPs as a potential prevention strategy for CVD via targeting the gut microbiota. Because of the large inter-individual differences in the composition and associated metabolism of the gut microbial community, a detailed structural characterization of MAPs along with gut microbiota and the effect of their metabolites on cardioprotective activity is necessary to design MAPs with potential use for the prevention and treatment of CVD.

## Figures and Tables

**Table 1:** Structural features and protective effects of polysaccharides from different marine algae.

Source	Polysaccharide Type	Main Composition	Linkage Units	Bioactive Activities	Refs
*Laminaria japonica*	Fucoidan	L-fucose	α-(1→2)-linked fucose or α-(1→3)-linked fucose	endothelial protective activity ↑	[18,19]
*Laminaria*, *Saccharina*, *Ascophyllum*, *Durvillaea*, *Macrocystis*, *Ecklonia*, and *Lessonia* spp.	Alginate	mannuronic acid, guluronic acid	(1,4)-linked β-D-mannuronic acid (ManA) and α-L-guluronic acid (GluA)	fasting blood glucose ↓, total cholesterol ↓, total-body fat ↓	[20,21]
*Gelidium* and *Gracilaria* spp.	Agar	agarose and agaropectin	3,6-anhydro-L-galactopyranose and D-galactose/L-galactose and D-galactose linked sulfate groups	antidiabetic effects ↑	[22,23]
*Porphyra* spp.	Porphyran	galactose, galactose 6-sulfate	(1→4)-linked α-L-galactose 6-sulfate units and (1→3)-linked β-D-galactose units	antihyperlipidemic activity ↑, antioxidant capacities ↑	[24]
*Eucheuma cottonii*, *Chondrus crispus*	Carrageenan	D-galactose, 3,6-anhydro-D-galactose	α-1,3-glucosidic and β-1,4-glycosidic linkages	total cholesterol ↓, low-density lipoprotein cholesterol ↓	[25,26]
*Ulva*, *Enteromorpha* spp.	Ulvan	rhamnose, L-rhamnose 3-sulfate	O-3-sulfate rhamnose and β-D-glucuronic acid(1→4)-L-rhamnose 3-sulfate, O-3-sulfate rhamnose and α-L-iduronic acid(1→4)-L-rhamnose 3-sulfate	antihyperlipidemic activity ↑	[27,28]
*Monostroma nitidum*	Sulfated rhamnan	rhamnose	→3)-α-L-Rhap-(1→ and →2)-α-L-Rhap-(1→ residues	thrombolytic activity ↑, antithrombotic activity ↑	[29,30,31]

## Data Availability

Not applicable.
